# Supplementary material for: Health status outcome among cannabis addicts after treatment of addiction
Source: PLoS One. 2023 Nov 27;18(11):e0290730. doi: 10.1371/journal.pone.0290730 (PMC10681311; doi:10.1371/journal.pone.0290730)
Supplement: Attachment — Submitted filename: reply.docx [file pone.0290730.s002.docx]

PONE-D-23-02435

Health status outcome among cannabis addicts after treatment of addiction

PLOS ONE

Dear Dr. Emara,

Thank you for submitting your manuscript to PLOS ONE. After careful consideration, we feel that it has merit but does not fully meet PLOS ONE’s publication criteria as it currently stands. Therefore, we invite you to submit a revised version of the manuscript that addresses the points raised during the review process.

Please submit your revised manuscript by Apr 30 2023 11:59PM. If you will need more time than this to complete your revisions, please reply to this message or contact the journal office at [plosone@plos.org](mailto:plosone@plos.org). When you're ready to submit your revision, log on to https://www.editorialmanager.com/pone/ and select the 'Submissions Needing Revision' folder to locate your manuscript file.

Please include the following items when submitting your revised manuscript:

- A rebuttal letter that responds to each point raised by the academic editor and reviewer(s). You should upload this letter as a separate file labeled 'Response to Reviewers'.

Thank you, I did (see below)

- A marked-up copy of your manuscript that highlights changes made to the original version. You should upload this as a separate file labeled 'Revised Manuscript with Track Changes'.

Thank you, I did

- An unmarked version of your revised paper without tracked changes. You should upload this as a separate file labeled 'Manuscript'.

  Thank you, I did

If you would like to make changes to your financial disclosure, please include your updated statement in your cover letter. Guidelines for resubmitting your figure files are available below the reviewer comments at the end of this letter.

If applicable, we recommend that you deposit your laboratory protocols in protocols.io to enhance the reproducibility of your results. Protocols.io assigns your protocol its own identifier (DOI) so that it can be cited independently in the future. For instructions see: <https://journals.plos.org/plosone/s/submission-guidelines#loc-laboratory-protocols>. Additionally, PLOS ONE offers an option for publishing peer-reviewed Lab Protocol articles, which describe protocols hosted on protocols.io. Read more information on sharing protocols at <https://plos.org/protocols?utm_medium=editorial-email&utm_source=authorletters&utm_campaign=protocols>.

We look forward to receiving your revised manuscript.

Kind regards,

Yasmina Abd‐Elhakim
Academic Editor
PLOS ONE

Dear Prof. Yasmina Abd‐Elhakim	(Editor)

Thanks for your effort and giving my opportunity to revise my article.

I reply to all questions of reviewers and editor. But I ready to reply to any question or correction in future.

Thank You

Ashraf Emara

**Journal Requirements:**

When submitting your revision, we need you to address these additional requirements.

1. Please ensure that your manuscript meets PLOS ONE's style requirements, including those for file naming. The PLOS ONE style templates can be found at 
https://journals.plos.org/plosone/s/file?id=wjVg/PLOSOne_formatting_sample_main_body.pdf and 
https://journals.plos.org/plosone/s/file?id=ba62/PLOSOne_formatting_sample_title_authors_affiliations.pdf

Thank you, I did

2. Thank you for stating the following financial disclosure: 
   "No fund"

At this time, please address the following queries:

a) Please clarify the sources of funding (financial or material support) for your study. List the grants or organizations that supported your study, including funding received from your institution. 
b) State what role the funders took in the study. If the funders had no role in your study, please state: “The funders had no role in study design, data collection and analysis, decision to publish, or preparation of the manuscript.”
c) If any authors received a salary from any of your funders, please state which authors and which funders.
d) If you did not receive any funding for this study, please state: “The authors received no specific funding for this work.”

### The researchers would like to thank the Deanship of Scientific Research, Qassim University for funding publication of this project.

Please include your amended statements within your cover letter; we will change the online submission form on your behalf.

**Thanks**

3. Thank you for stating the following in the Acknowledgments Section of your manuscript: 
    "The researchers would like to thank the Deanship of Scientific Research, Qassim University 
for funding publication of this project."

We note that you have provided funding information that is not currently declared in your Funding Statement. However, funding information should not appear in the Acknowledgments section or other areas of your manuscript. We will only publish funding information present in the Funding Statement section of the online submission form. 
Please remove any funding-related text from the manuscript and let us know how you would like to update your Funding Statement. Currently, your Funding Statement reads as follows: 
     "No fund"

Please include your amended statements within your cover letter; we will change the online submission form on your behalf.

Thank you, I remove it

4. In your Data Availability statement, you have not specified where the minimal data set underlying the results described in your manuscript can be found. PLOS defines a study's minimal data set as the underlying data used to reach the conclusions drawn in the manuscript and any additional data required to replicate the reported study findings in their entirety. All PLOS journals require that the minimal data set be made fully available. For more information about our data policy, please see http://journals.plos.org/plosone/s/data-availability.

"Upon re-submitting your revised manuscript, please upload your study’s minimal underlying data set as either Supporting Information files or to a stable, public repository and include the relevant URLs, DOIs, or accession numbers within your revised cover letter. For a list of acceptable repositories, please see http://journals.plos.org/plosone/s/data-availability#loc-recommended-repositories. Any potentially identifying patient information must be fully anonymized.

Important: If there are ethical or legal restrictions to sharing your data publicly, please explain these restrictions in detail. Please see our guidelines for more information on what we consider unacceptable restrictions to publicly sharing data: http://journals.plos.org/plosone/s/data-availability#loc-unacceptable-data-access-restrictions. Note that it is not acceptable for the authors to be the sole named individuals responsible for ensuring data access.

We will update your Data Availability statement to reflect the information you provide in your cover letter.

The original contributions presented in the study are included in the article/supplementary material, further inquiries can be directed to the corresponding author.

5. Your ethics statement should only appear in the Methods section of your manuscript. If your ethics statement is written in any section besides the Methods, please move it to the Methods section and delete it from any other section. Please ensure that your ethics statement is included in your manuscript, as the ethics statement entered into the online submission form will not be published alongside your manuscript.

Moved to the method section.

**Reviewers' comments:**

Reviewer #1: Interesting study entitled "Health status outcome among cannabis addicts after treatment of addiction".

This is a very interesting study as it is the first study to evaluate the impact on health status after treatment of cannabis addiction.
The manuscript is written with attractive and novel results for the scientific community.
If the authors complete the analyzes requested below to strengthen the results, this paper could be a very strong piece of work that could be accepted for publication in PLOS ONE.

However, some elements need to be addressed by the authors to help improve the quality of the manuscript:

**MAJOR COMMENTS:**

1) In methods, can you provide what treatment patients received, and mention in the discussion and limitations if it could interact with the samples blood test performed in this study.

2) Please provide the number of lost of follow up.
Thank you, I agree

The treatment patients received is mentioned under method section.

## **Treatment patients received**

Psychotherapy and medication are used to treat cannabis use disorder. The majority of its attention is given to psychotherapy techniques like motivational enhancement treatment, cognitive behavioral therapy, and contingency management. Psychosocial therapy has occasionally been combined with pharmaceutical treatments. Pharmacotherapy aimed to reduce withdrawal symptoms such agitation, anxiety, restlessness, decreased appetite, and erratic sleeping patterns. It contains lofexidine, baclofen, zolpidem, and quetiapine Patients were followed up with once a week.

3)Concerning figure and tables:
- consider to add a flowchart

Thank you, I agree

Stage I

Medical examination

100 male cannabis abusers

20 male healthy people [control group]

Blood sample at admission

Stage II

Treatment for cannabis abuse

Stage III

Blood sample after complete cure from abuse for investigation

Results were tabulated, analyzed and discussed.

Follow up

**Figure 1**: Schematic diagram of the study design

- Please in figure 1, 2, 3, 4, please provide p-value between the different columns. A table with all the data summarized with 4 columns should be considered (Control, Cannabis users, cannabis users after treatment, and p-value)

Thank you, I agree

Table 5: Body mass index, vital signs, Liver function tests, blood glucose level, kidney function tests and oxidative stress biomarkers for the control and cannabis groups [n=120] before and after treatment.

| **Items** | **Group I**  **[n=20]** | **Group II [n=100]** | | **P-value** |
| --- | --- | --- | --- | --- |
|  |  | Before treatment | After treatment |  |
| Body Mass Index [kg/m^2^] | 25.09 ± 1.16 | 25.07 ± 2.12 | 24.69 ± 2.10 | 0.385 |
| Temperature [°C] | 36.76 ± 1.16 | 36.86 ± 1.24 | 36.68 ± 1.18^b^ | 0.574 |
| Heart Rate [bpm] | 75.50 ± 2.93 | 97.60 ± 9.51^a^ | 85.06 ± 5.25^ab^ | ≤0.001^#^ |
| Systolic Blood Pressure [mmHg] | 123.90 ± 3.29 | 135.59 ± 8.56^a^ | 123.21 ± 6.20^b^ | ≤0.001^#^ |
| Diastolic Blood Pressure [mmHg] | 80.20 ± 3.04 | 87.64 ± 2.78^a^ | 83.87 ± 2.96^ab^ | ≤0.001^#^ |
| Respiratory Rate [bpm] | 19.80 ± 1.24 | 22.36 ± 1.65^a^ | 19.75 ± 1.53^b^ | ≤0.001^#^ |
| Albumin [g/dL] | 4.46 ± 0.21 | 4.78 ± 0.55^a^ | 4.40 ± 0.53^b^ | ≤0.001^#^ |
| Total bilirubin [mg/dL] | 0.92 ± 0.07 | 1.46 ± 0.36^a^ | 0.96 ± 0.21^b^ | ≤0.001^#^ |
| Direct bilirubin [mg/dL] | 0.15 ± 0.07 | 0.23 ± 0.07^a^ | 0.14 ± 0.08^b^ | ≤0.001^#^ |
| Aspartate aminotransferase [U/L] | 20.80 ± 5.80 | 32.57 ± 8.31^a^ | 22.98 ± 3.82^b^ | ≤0.001^#^ |
| Alanine aminotransferase [U/L] | 20.25 ± 5.95 | 32.59 ± 8.24^a^ | 23.24 ± 8.26^b^ | ≤0.001^#^ |
| Alkaline phosphatase [U/L] | 74.30 ± 18.24 | 90.30 ± 20.31^a^ | 81.85 ± 25.76^b^ | 0.003 |
| Fasting blood glucose level [mg/dL] | 88.00 ± 4.21 | 97.17 ± 13.71^a^ | 84.78 ± 9.24^b^ | ≤0.001^#^ |
| Urea [mg/dL] | 30.33 ± 6.01 | 30.14 ± 8.59 | 30.60 ± 7.89 | 0.922 |
| Creatinine [mg/dL] | 0.90 ± 0.08 | 1.03 ± 0.20^a^ | 1.02 ± 0.16^a^ | 0.009 |
| Thiobarbituric acid-reactive substances [nmol/ml] | 1.23 ± 0.12 | 2.45 ± 0.17^a^ | 2.43 ± 0.18^a^ | ≤0.001^#^ |
| Reduced glutathione [ng/ml] | 47.38 ± 2.21 | 28.63 ± 4.45^a^ | 28.62 ± 3.77^a^ | ≤0.001^#^ |
| Superoxide dismutase [ng/ml] | 0.82 ± 0.08 | 1.96 ± 0.09^a^ | 1.94 ± 0.12^a^ | ≤0.001^#^ |
| Catalase [ng/L] | 72.15 ± 2.22 | 57.25 ± 2.11^a^ | 56.53 ± 4.37^a^ | ≤0.001^#^ |

Group I – Control group; Group II – Cannabis group, Values expressed as Mean ± SD. ^a^ Significant compared with control group.

^b^ Significant compared with before treatment	Significant at p<0.05 level.

- In table 5, 6, 7 and 8, authors mentions a correlation between CWAS, cannabis level, age, and duration of cannabis intake, BMI, and vitals signes. It is unclear how variables are corelate. Unless I’m mistaken, Person correlation variable under 0.5-0.7 is not a positive correlation, even if p-value is significant. There is no p-value needed in this context.
- It could be interesting to perform some subgroups analysis (in younger and older patients for example)

Thank you, I agree

**We perform dividing the patients into:**

## 1. **Correlations of the studied parameters for young patients [aged 18 to 35 years old]**

## 2. **Correlations of the studied parameters for patients aged more 35 years old**

3. Table 6: Pearson correlation coefficients between Cannabis Withdrawal Assessment Scale [CWAS], cannabis level, Age and duration of Cannabis intake, Body Mass Index, and Vital Signs in the cannabis group at admission [AA] and after the treatment [AT] ] for patients aged 18 to 35 years old, [n=59].

| **Items** | **CWAS** | | **9-THC level** | | **Age** | | **Duration of cannabis intake** | |
| --- | --- | --- | --- | --- | --- | --- | --- | --- |
|  | **AA** | AT | **AA** | AT | **AA** | AT | **AA** | AT |
| **BMI [kg/m^2]^** | 0.099 | 0.125 | 0.087 | 0.083 | 0.096 | 0.045 | 0.067 | 0.098 |
| **Temp. [°C]** | 0.043 | 0.098 | 0.078 | 0.055 | 0.046 | 0.076 | 0.052 | 0.049 |
| **HR [bpm]** | 0.082 | 0.262 | 0.793** | 0.110 | 0.098 | 0.065 | 0.578** | 0.048 |
| **SBP [mmHg]** | 0.134 | 0.017 | 0.728** | 0.146 | 0.698** | 0.079 | 0.799** | 0.112 |
| **DBP [mmHg]** | 0.067 | 0.092 | 0.056 | 0.076 | 0.077 | 0.098 | 0.069 | 0.066 |
| **RR [bpm]** | 0.067 | 0.093 | 0.083 | 0.089 | 0.056 | 0.058 | 0.089 | 0.049 |

4. CWAS - Cannabis Withdrawal Assessment Scale; 9-THC- tetrahydrocannabinol; Temp- Temperature; BMI- Body Mass Index; HR- Heart Rate; SBP – Systolic Blood Pressure; DBP – Diastolic Blood Pressure; RR- Respiratory Rate.
5. * Correlation was statistically significant at 0.05 level [2-tailed].
6. ** Correlation was statistically significant at 0.01 level [2-tailed].

## 7. Correlations of the studied parameters for young patients [aged 18 to 35 years old]

### 8. Body Mass Index [BMI]

9. Table [6] revealed that there were no significant correlations in group II at admission and after treatment between BMI and CWAS, tetrahydrocannabinol level, age and duration of cannabis intake.

### 10. Vital signs

11. Table [6] revealed that body temperature had no significant correlation in group II before and after treatment with CWAS, tetrahydrocannabinol level, age, and the duration of cannabis intake.
12. Table [6] revealed that HR had no significant correlation in group II at admission and after treatment with CWAS and age. While HR showed significant correlation in group II at admission with tetrahydrocannabinol level and the duration of cannabis intake and no significant correlation in group II after treatment with tetrahydrocannabinol level and the duration of cannabis intake.
13. Table [6] revealed that SBP had significant correlations in group II at admission with tetrahydrocannabinol level, age, and the duration of cannabis intake and no significant correlations after treatment. Whereas, SBP showed no significant correlation in group II at admission and after treatment with CWAS.
14. Table [6] revealed that DBP had no significant correlation in group II at admission and after treatment with CWAS, tetrahydrocannabinol level, age, and duration of cannabis intake.
15. Table [6] revealed that there were no significant correlations in group II at admission and after treatment between RR and CWAS, tetrahydrocannabinol level, age, and duration of cannabis intake.
17. Table 7: Pearson correlation coefficients between Cannabis Withdrawal Assessment Scale [CWAS], tetrahydrocannabinol level, Age, duration of Cannabis intake, liver function tests values, and blood glucose level in the cannabis group at admission [AA] and after the treatment [AT] ] for patients aged 18 to 35 years old, [n=59].

| **Items** | **CWAS** | | **9-THC  level** | | **Age** | | **Duration of cannabis intake** | |
| --- | --- | --- | --- | --- | --- | --- | --- | --- |
|  | AA | AT | AA | AT | AA | AT | AA | AT |
| **Albumin [g/dL]** | 0.099 | 0.076 | 0.088 | 0.067 | 0.058 | 0.049 | 0.099 | 0.086 |
| **Total bilirubin [mg/dL]** | 0.082 | 0.079 | 0.096 | 0.068 | 0.034 | 0.047 | 0.067 | 0.084 |
| **Direct bilirubin [mg/dL]** | 0.045 | 0.055 | 0.078 | 0.089 | 0.094 | 0.088 | 0.064 | 0.062 |
| **AST [U/L]** | 0.056 | 0.052 | 0.720^**^ | 0.112 | 0.114 | 0.133 | 0.510* | 0.101 |
| **ALT [U/L]** | 0.122 | 0.048 | 0.778^**^ | 0.126 | 0.046 | 0.054 | 0.599** | 0.076 |
| **ALP [U/L]** | 0.054 | 0.066 | 0.110 | 0.119 | 0.077 | 0.098 | 0.069 | 0.100 |
| **Blood glucose level [mg/dL]** | 0.086 | 0.098 | 0.105 | 0.100 | 0.079 | 0.048 | 0.058 | 0.089 |

18. CWAS- Cannabis Withdrawal Assessment Scale; 9-THC- tetrahydrocannabinol; AST- Aspartate aminotransferase; ALT- Alanine aminotransferase; ALP- Alkaline phosphatase.
19. * Correlation was statistically significant at 0.05 level [2-tailed].
20. ** Correlation was statistically significant at 0.01 level [2-tailed].

### 21. Liver function tests

22. Table [7] revealed that there were no significant correlations in group II at admission and after treatment between albumin and CWAS, tetrahydrocannabinol level, age, and the duration of cannabis intake.
23. Table [7] revealed that there were no significant correlations in group II at admission and after treatment between total and direct bilirubin and CWAS, tetrahydrocannabinol level, age, and the duration of cannabis intake.
24. Table [7] revealed that there were significant correlations in group II at admission between aspartate aminotransferase and alanine aminotransferase and tetrahydrocannabinol level and the duration of cannabis intake. On the other hand, they showed no significant correlations after treatment. While, they showed no significant correlations with CWAS and age at admission and after treatment.
25. Table [7] revealed that there were no significant correlations in group II at admission and after treatment between alkaline phosphatase and CWAS, tetrahydrocannabinol level, age, and the duration of cannabis intake.
26. Table [7] revealed that there were significant correlations in group II at admission and after treatment between blood glucose level and CWAS, tetrahydrocannabinol level, age, and duration of cannabis intake.
27. Table 8: Pearson correlation coefficients between Cannabis Withdrawal Assessment Scale [CWAS], tetrahydrocannabinol level, Age, duration of Cannabis intake, renal function tests values in the cannabis group at admission [AA] and after the treatment [AT] ] for patients aged 18 to 35 years old, [n=59].

| **Items** | **CWAS** | | **9-THC level** | | **Age** | | **Duration of cannabis intake** | |
| --- | --- | --- | --- | --- | --- | --- | --- | --- |
|  | AA | AT | AA | AT | AA | AT | AA | AT |
| **Urea [mg/dL]** | 0.121 | 0.111 | 0.067 | 0.099 | 0.114 | 0.115 | 0.088 | 0.076 |
| **Creatinine [mg/dL]** | 0.043 | 0.034 | 0.055 | 0.064 | 0.039 | 0.046 | 0.052 | 0.064 |

28. CWAS -Cannabis Withdrawal Assessment Scale; 9-THC- tetrahydrocannabinol.
29. * Correlation was statistically significant at 0.05 level [2-tailed].
30. ** Correlation was statistically significant at 0.01 level [2-tailed].
31. Table [8] revealed that there were no significant correlations in group II at admission and after treatment between urea and CWAS, tetrahydrocannabinol level, age, and duration of cannabis intake.
32. Table [8] revealed that there were no significant correlations in group II at admission and after treatment between creatinine and CWAS, tetrahydrocannabinol level, age, and duration of cannabis intake.
33. Table 9: Pearson correlation coefficients between CWAS, tetrahydrocannabinol level, Age, duration of Cannabis, enzymatic antioxidants, and lipid peroxidation in the cannabis group at admission [AA] and after the treatment [AT] ] for patients aged 18 to 35 years old, [n=59].

| **Items** | **CWAS** | | **9-THC level** | | **Age** | | **Duration of cannabis intake** | |
| --- | --- | --- | --- | --- | --- | --- | --- | --- |
|  | AA | AT | AA | AT | AA | AT | AA | AT |
| **TBARS** | 0.111 | 0.091 | 0.708** | 0.101 | 0.069 | 0.099 | 0.858** | 0.098 |
| **GSH [mg/dl]** | -0.004 | -0.071 | -0.657** | -0.117 | -0.112 | -0.057 | -0.718** | -0.109 |
| **SOD [ng/ml]** | 0.048 | 0.067 | 0.625** | 0.095 | 0.096 | 0.078 | 0.365** | 0.065 |
| **CAT [ku/l]** | -0.098 | -0.112 | - 0.546** | -0.112 | -0.078 | -0.087 | -0.495** | -0.088 |

34. CWAS -Cannabis Withdrawal Assessment Scale; 9-THC- tetrahydrocannabinol; TBARS – Thiobarbituric acid-reactive substances; GSH – Reduced glutathione; SOD – Superoxide dismutase; CAT – Catalase.
35. * Correlation was statistically significant at 0.05 level [2-tailed].
36. ** Correlation was statistically significant at 0.01 level [2-tailed].
37. Table [9] revealed that there were significant correlations in group II at admission between oxidative stress markers [Thiobarbituric acid-reactive substances, reduced glutathione, superoxide dismutase and catalase] and CWAS, tetrahydrocannabinol level, age, and the duration of cannabis intake. On the other hand, they showed no significant correlations after treatment. While, they showed no significant correlations with CWAS and age at admission and after treatment.

## 39. Correlations of the studied parameters for patients aged more 35 years old

40. Table 10: Pearson correlation coefficients between Cannabis Withdrawal Assessment Scale [CWAS], cannabis level, Age and duration of Cannabis intake, Body Mass Index, and Vital Signs in the cannabis group at admission [AA] and after the treatment [AT] for patients aged more than 35 years old, [n=41].

| **Items** | **CWAS** | | **9-THC level** | | **Age** | | **Duration of cannabis intake** | |
| --- | --- | --- | --- | --- | --- | --- | --- | --- |
|  | AA | AT | AA | AT | AA | AT | AA | AT |
| **BMI [kg/m^2]^** | 0.089 | 0.125 | 0.055 | 0.045 | 0.067 | 0.062 | 0.**6**89** | 0.576** |
| **Temp. [°C]** | 0.082 | 0.111 | 0.119 | 0.067 | 0.083 | 0.084 | 0.092 | 0.043 |
| **HR [bpm]** | 0.082 | 0.262 | 0.793** | 0.266* | 0.749** | 0.386** | 0.454** | 0.267* |
| **SBP [mmHg]** | 0.124 | 0.099 | 0.896** | 0.256* | 0.844** | 0.239* | 0.779** | 0.355** |
| **DBP [mmHg]** | 0.087 | 0.098 | 0.115 | 0.053 | 0.114 | 0.067 | 0.118 | 0.052 |
| **RR [bpm]** | 0.056 | 0.092 | 0.085 | 0.037 | 0.056 | 0.067 | 0.754** | 0.398** |

41. CWAS - Cannabis Withdrawal Assessment Scale; 9-THC- tetrahydrocannabinol; Temp- Temperature; BMI- Body Mass Index; HR- Heart Rate; SBP – Systolic Blood Pressure; DBP – Diastolic Blood Pressure; RR- Respiratory Rate.
42. * Correlation was statistically significant at 0.05 level [2-tailed].
43. ** Correlation was statistically significant at 0.01 level [2-tailed].

### 44. Body Mass Index [BMI]

45. Table [10] revealed that there were no significant correlations in group II at admission and after treatment between BMI and age, duration of cannabis intake and the tetrahydrocannabinol level. On the other hand, the present study revealed that there were significant correlations in group II at admission and after treatment between BMI and duration of cannabis intake.

### 46. Vital signs

47. Table [10] revealed that body temperature had no significant correlation in group II before and after treatment with CWAS, tetrahydrocannabinol level, age, and duration of cannabis intake.
48. Table [10] revealed that HR had no significant correlation in group II at admission and after treatment with CWAS. While, HR showed significant correlations in group II at admission and after treatment with tetrahydrocannabinol level, age and the duration of cannabis intake.
49. Table [10] revealed that SBP had no significant correlations in group II at admission and after treatment with CWAS. Whereas, SBP showed significant correlations in group II at admission and after treatment with the tetrahydrocannabinol level, age and the duration of cannabis intake.
50. Table [10] revealed that DBP had no significant correlation in group II at admission and after treatment with CWAS, tetrahydrocannabinol level, age, and duration of cannabis intake.
51. Table [10] revealed that there were significant correlations in group II at admission and after treatment between RR and the duration of cannabis intake. On the other hand, the present study revealed that there were no significant correlations in group II at admission and after treatment between RR and the tetrahydrocannabinol levels, CWAS and age.
53. Table 11: Pearson correlation coefficients between Cannabis Withdrawal Assessment Scale [CWAS], tetrahydrocannabinol level, Age, duration of Cannabis intake, liver function tests values, and blood glucose level in the cannabis group at admission [AA] and after the treatment [AT] for patients aged more than 35 years old, [n=41].

| **Items** | **CWAS** | | **9-THC level** | | **Age** | | **Duration of cannabis intake** | |
| --- | --- | --- | --- | --- | --- | --- | --- | --- |
|  | AA | AT | AA | AT | AA | AT | AA | AT |
| **Albumin [g/dL]** | 0.129 | 0.166 | 0.091 | 0.026 | 0.067 | 0.092 | 0.127 | 0.158 |
| **Total bilirubin [mg/dL]** | 0.082 | 0.079 | 0.106 | 0.099 | 0.021 | 0.029 | 0.089 | 0.094 |
| **Direct bilirubin [mg/dL]** | 0.023 | 0.007 | 0.115 | 0.071 | 0.102 | 0.082 | 0.085 | 0.042 |
| **AST [U/L]** | 0.094 | 0.105 | 0.620^**^ | 0.205* | 0.512** | 0.200* | 0.710** | 0.398** |
| **ALT [U/L]** | 0.191 | 0.142 | 0.479^**^ | 0.321^**^ | 0.381** | 0.233* | 0.679** | 0.359** |
| **ALP [U/L]** | 0.093 | 0.094 | 0.110 | 0.119 | 0.078 | 0.102 | 0.108 | 0.131 |
| **Blood glucose level [mg/dL]** | 0.106 | 0.094 | 0.605** | 0.200* | 0.673** | 0.356** | 0.648** | 0.383** |

55. CWAS- Cannabis Withdrawal Assessment Scale; 9-THC- tetrahydrocannabinol; AST- Aspartate aminotransferase; ALT- Alanine aminotransferase; ALP- Alkaline phosphatase.
56. * Correlation was statistically significant at 0.05 level [2-tailed].
57. ** Correlation was statistically significant at 0.01 level [2-tailed].

### 58. Liver function tests

59. Table [10] revealed that there were no significant correlations in group II at admission and after treatment between albumin and CWAS, tetrahydrocannabinol level, age, and the duration of cannabis intake.
60. Table [10] revealed that there were no significant correlations in group II at admission and after treatment between total and direct bilirubin and CWAS, the tetrahydrocannabinol levels, age, and duration of cannabis intake.
61. Table [10] revealed that there were significant correlations in group II at admission and after treatment between liver enzymes [aspartate aminotransferase and alanine aminotransferase] and the tetrahydrocannabinol level, age, and the duration of cannabis intake. On the other hand, they showed no significant correlations in group II at admission and after treatment with CWAS. The current study revealed that there were no significant correlations in group II at admission and after treatment between alkaline phosphatase and CWAS, tetrahydrocannabinol levels, age, and duration of cannabis intake.
62. Table [10] revealed that there were significant correlations in group II at admission and after treatment between blood glucose level and tetrahydrocannabinol level, age, and duration of cannabis intake. On the other hand, it showed no significant correlations in group II at admission and after treatment with CWAS.
63. Table 11: Pearson correlation coefficients between Cannabis Withdrawal Assessment Scale [CWAS], tetrahydrocannabinol level, Age, duration of Cannabis intake, renal function tests values in the cannabis group at admission [AA] and after the treatment [AT] for patients aged more than 35 years old, [n=41].

| **Items** | **CWAS** | | **9-THC level** | | **Age** | | **Duration of cannabis intake** | |
| --- | --- | --- | --- | --- | --- | --- | --- | --- |
|  | AA | AT | AA | AT | AA | AT | AA | AT |
| **Urea [mg/dL]** | 0.131 | 0.161 | 0.084 | 0.100 | 0.145 | 0.142 | 0.096 | 0.097 |
| **Creatinine [mg/dL]** | 0.029 | 0.028 | -0.048 | -0.034 | 0.035 | 0.025 | 0.047 | 0.058 |

65. CWAS -Cannabis Withdrawal Assessment Scale; 9-THC- tetrahydrocannabinol.
66. * Correlation was statistically significant at 0.05 level [2-tailed].
67. ** Correlation was statistically significant at 0.01 level [2-tailed].
68. Table [8] revealed that there were no significant correlations in group II at admission and after treatment between urea and CWAS, tetrahydrocannabinol level, age, and duration of cannabis intake.
69. Table [8] revealed that there were no significant correlations in group II at admission and after treatment between creatinine and CWAS, tetrahydrocannabinol level, age, and duration of cannabis intake.
70. Table 12: Pearson correlation coefficients between CWAS, tetrahydrocannabinol level, Age, duration of Cannabis, enzymatic antioxidants, and lipid peroxidation in the cannabis group at admission [AA] and after the treatment [AT] for patients aged more than 35 years old, [n=41].

| **Items** | **CWAS** | | **9-THC level** | | **Age** | | **Duration of cannabis intake** | |
| --- | --- | --- | --- | --- | --- | --- | --- | --- |
|  | AA | AT | AA | AT | AA | AT | AA | AT |
| **TBARS** | 0.108 | 0.031 | 0.501** | 0.201* | 0.566** | 0.301** | 0.898** | 0.389** |
| **GSH [mg/dl]** | -0.004 | -0.071 | -0.627* | -0.227* | -0.724** | -0.457** | -0.614** | -0.409** |
| **SOD [ng/ml]** | 0.015 | 0.014 | 0.729** | 0.405** | 0.662* | 0.302** | 0.557** | 0.334** |
| **CAT [ku/l]** | -0.058 | -0.183 | - 0.621** | -0.256* | 0.655** | -0.254* | -0.675** | -0.305** |

71. CWAS -Cannabis Withdrawal Assessment Scale; 9-THC- tetrahydrocannabinol; TBARS – Thiobarbituric acid-reactive substances; GSH – Reduced glutathione; SOD – Superoxide dismutase; CAT – Catalase.
72. * Correlation was statistically significant at 0.05 level [2-tailed].
73. ** Correlation was statistically significant at 0.01 level [2-tailed].

3) At the beginning of the discussion, authors usually summarized the majors findings and results of the study. This work evaluates biological and vital signs of an intervention which is the treatment of cannabis addiction. Please, choose the most important results to prove your point.

Thank you, I agree

This work evaluates biological (including oxidative stress) markers and vital signs of an intervention which is the treatment of cannabis addiction. It demonstrated that most of biological and vital signs alterations are resolved in comparison with control group. While, oxidative stress markers were still high.

3) This study about cannabis and his pleiotropic effect on human body is very interesting. But the second part of the study is also assessing the impact of the treatment of the addiction. Do you have a proof of the weaning of the patients by a scale?
Thank you, I agree

During the follow up, the screening and routine medical analysis is performed.

4) Authors didn’t address the limitations of the study in the discussion.

Thank you, I agree

# Limitations of the study

The cross-sectional design of the study, together with the limited sample size and short participant follow-up duration, were its limitations. This project only had male participants. By working in conjunction with the Drug Control Department at the Ministry of Interior and adhering to the essential criteria, it requires longitudinal research with additional inspection and confirmation of the addictive components. After then, a group of addicts will be treated with a long-term program through highly specialist treatment facilities.

5) Authors didn’t discuss about the significant difference of level of oxidative stress biomarker between the 2 groups at the admission and then didn’t normalize after treatment in groupe 2.

Numerous cellular antioxidant enzymes and compounds protect cells from the damaging effects of oxidative stress. These include enzyme-based antioxidants like superoxide dismutase, catalase, and glutathione peroxidase as well as non-enzymatic antioxidants like vitamin C, vitamin E, and glutathione [44]. Oxidative stress is defined as the presence of excessive free radical formation that exceeds the capacity of the cellular antioxidant system, resulting in damage to membrane lipids (lipid peroxidation), as well as the oxidation of intracellular enzyme proteins and nucleic acids [45]. Mitochondrial malfunction is the main source of free radicals that cause oxidative stress [46]. It has been noted that cannabis can cause mitochondrial malfunction [47]. ROS were significantly produced as a result of THC. Cannabis-related mitochondrial dysfunction may lead to an increase in H2O2 production since mitochondria are both sources and targets of ROS. The free radical leak increased as a result of THC exposure, boosting the respiratory chain's fraction of electrons that convert O2 to ROS [48]. In 2017, Bayazit et al. discovered higher levels of total oxidant status, oxidative stress index, and interleukin [IL] 1, IL-6, IL-8, and tumor necrosis factor [TNF] in cannabis-dependent individuals compared to healthy individuals [49]. In 2020, Guler et al. concluded that SC, in addition to its potential for DNA damage, could cause an increase in oxidative stress and inflammatory processes [50].  In 2016, Nagy et al. discovered a significant rise in both TBARS and protein oxidation in heroin and cannabis male and female addicts in comparison to the control group. They concluded that cannabis addiction had harmful effects on the immune system by affecting oxidative stress and ROS release [51]. These data are consistent with the findings of this study. The present study demonstrated a significant increase in the mean thiobarbituric acid-reactive substances, superoxide dismutase values and decrease in the mean reduced glutathione and catalase values in cannabis user group at admission compared to healthy controls. These findings agree with a previous report showing enhanced oxidative stress markers after use of cannabis [52]. Also, Cerretani et al., 2020 reported that reduced glutathione/oxidized glutathione ratio and catalase activity was significantly reduced and increase in malondialdehyde content after exposure to cannabis [53]. On the other hand, in 2021, Dawidowicz et al. found that THC has antioxidant properties similar to vitamins E and C, scavenging free radicals, reducing metal ions, and protecting oxidation processes [54]. Our study notes that the dose of cannabis used has not been reported as well as the timing of measurement is different in our study. As a result, various cellular adaptations are very likely to occur. According to Bani-Ahmad, there was no discernible difference between the control group and the hashish users in terms of TBARS content. The low levels of oxidative markers found in hashish addicts may be due to a number of variables that relate to different facets of hashish cultivation, preparation, and pharmacokinetics when used orally. These elements taken together may contribute to low and unexpected plasma levels of powerful THC metabolites [55].

6) In the conclusion, authors should be more precise and factual about the findings.
Thank you, I agree

The current findings show that cannabis addiction harms various body organs except kidneys and has serious consequences on addicts' health. This study revealed that after treatment, there was no improvement in oxidative stress biomarker values, but there was an improvement in the other biological parameters.

MINOR COMMENTS:

4) In table 1 legend, I didn’t understand why you referred to alcohol group ? Is-it a mistake ?

Thank you, I agree

It is corrected

5) The authors should use a native english speaker to correct minor grammatical mistakes.

Thank you, I agree

It is corrected

8) I think it would be easier to read if the authors rename groupe 1 and 2 with control groupe and cannabis user group.

Thank you, I agree

**I did this modification**

Reviewer #2: This manuscript describes a study that evaluated changes in health status before and after treatment using body mass index, glycaemic status, liver function, renal function, and oxidative stress in cannabis addicts.
The study was carried out on 100 hospitalized patients receiving addiction treatment and 20 healthy volunteers. Pre- and post-treatment health status indicators were determined by using cannabis level in urine, Body Mass Index, vital signs, some biochemical and oxidative stress parameters. While no change was observed in the BMI values and oxidative stress parameters between the groups before and after the treatment, it was revealed that there was a significant decrease in biochemical parameters in the dependent group after the treatment. Current findings reveal that cannabis addiction damages various body systems and has significant effects on the health status of the addict. This is an interesting finding that could lead to further research, confirming the phenomenon and attempting to identify the cause.
It is seen that the differences in the data obtained especially in the discussion section of the article are not fully revealed. The data obtained while writing the discussion and articles about it were written. However, as the last sentence, the author's opinion on why this result may have occurred has not been added. For this reason, I think that eliminating the deficiencies given below in detail will be important for the quality of the article.
Overall the study appears to have been conducted appropriately and results interpreted correctly (with some exceptions mentioned below). The article reads well, but there are some deficiencies that need to be edited from beginning to end, as there are places that cause misunderstanding at some points. The fact that this study lasted for two years raises fundamental concerns about the rationale for this study. Specific concerns that need to be addressed are as follows:

1. While it was stated that the dependent group was selected from male members, gender was not specified in the control group.

Thank you, I agree

We did in methodology section.

2. It should be scientifically stated why a two-year period was chosen to continue this study.

Thank you, I agree

Two-year period, to be sure of weaning from cannabis use

3. It was stated that the samples were collected both at the time of application and after the treatment. It is a matter of curiosity whether any measures have been taken to prevent this interval (approximately 2 years) from causing differences in the test results.

Thank you, I agree

The sample withdrawn immediately after finish the treatment, after that the patient under the follow up period.

4. In particular, how to distinguish whether the differences in the results obtained from the evaluation methods used in the material method section over the years are due to the treatment or to normal physiological processes.

Thank you, I agree

The sample withdrawn immediately after finish the treatment.
5. "Males were more likely than females to abuse substances, and most began using substances in high school." How did you come to this conclusion?

Thank you, I agree

This conclusion obtained from previous studies and we delete it.

6. "Compared to the control group, current results do not show a significant reduction in BMI at admission and after treatment." Can you explain why?

Thank you, I agree

Energy uptake, storage, and conservation are primarily mediated by CB1 receptors. Through a number of techniques, it works to maximize energy uptake and conservation. Food is made more palatable by modulating taste and smell pathways by stimulating CB1 receptors [33]. There is a lot of proof that THC or cannabis use causes the CB1R to be downregulated. Regular cannabis usage is linked to CB1R downregulation and desensitization, and CB1R levels stay low for 3–4 weeks after use has stopped [34]. During this time, recently abstinent users would have decreased hunger and elevated metabolic rates. They won't, however, continue to have short-term stimulation of appetite, increased energy consumption, and decreased metabolic rates. Therefore, until CB1R levels reach those from before cannabis usage, weight won't rise because energy intake and storage are still suppressed and metabolism is increased [35]. Ngueta et al., 2015 reported that a higher metabolic rate in marijuana users explain the lack of association between cannabis use and BMI, despite a high energy diet [36]. These data explained the findings of the current study. As compared to the control group, the current results do not indicate a significant reduction in BMI at admission and after treatment.

7. Could you indicate the reason why the vital signs in Group II were significantly lower in the discussion section?

Thank you, I agree

Immediately following marijuana use, the heart rate and blood pressure may increase [35]. Increased sympathetic nervous system activity is responsible for the increased heart rate, supine systolic and diastolic blood pressures, and forearm blood flow caused by marijuana cigarettes [36]. After a few repeated dosages, tolerance to several of these effects becomes noticeable. After just a day or two of repeated exposure, there may be significant tolerance to changes in blood pressure and elevated heart rate. Tolerance is lost just as quickly when marijuana or THC use is discontinued. Blood pressure falls and the resting heart rate drops after a few days of frequent oral THC exposure or a marijuana plant extract [37].

8. It is predicted that the fluctuation in blood glucose level occurs.

Thank you, I agree

The present study demonstrated that, compared to the control group, there was a significant increase in the mean fasting blood glucose level values of cannabis user group at admission but no significant change in cannabis user group after treatment. After treatment, the mean fasting blood glucose levels in cannabis user group decreased significantly compared to their respective at admission values. Marijuana use was linked to lower levels of fasting insulin and homeostasis model assessment of insulin resistance [37]. Cannabis use for 12 months is linked to nearly double the risk of diabetic ketoacidosis in one study of adults with T1D when compared to no cannabis use [38]. The variations in cannabis strain potencies may help to explain the variations in blood glucose levels. The potent preparations ('ganja' and 'charas') suppress appetite whereas the weak preparation ('bhang') induces anorexia. THC levels in peripheral blood in people who have smoked one or more marijuana cigarettes have exceeded the range of 5 to 250 ng/ml, whereas intravenous delivery of 10 mg/kg THC produced blood levels of 720 ng/ml after 20 minutes [9]. Other research has demonstrated that cannabis influence the stress response and increase the release of adrenocorticotropin (ACTH) [39, 40].

9. “Our findings found that in comparison to the control group, there was a significant increase in the mean levels of albumin, total bilirubin, direct bilirubin, AST, ALT, and ALP in group II at admission, but no significant change in group II after treatment”. Please explain why this situation has arisen.

Thank you, I agree

Hepatotoxicity is a possible side effect of using illegal drugs, such as marijuana. A study by Borini et al. evaluating clinical and laboratory changes in chronic marijuana users showed about fifty percent of patients have enlarged spleen or liver or both. Also, marijuana use has been linked to an increase in serum levels of AST, ALT, and AP, regardless of the amount or duration of use [40]. Our findings found that in comparison to the control group, there was a significant increase in the mean levels of albumin, total bilirubin, direct bilirubin, AST, ALT, and ALP in cannabis user group at admission, but no significant change in cannabis user group after treatment. These findings are consistent with a study by Devinsky et al. from 2018 which discovered that liver enzymes increased in chronic cannabis users and returned to normal following the drug's administration [41]. Additionally, Wani et al., 2017 found that cannabis users had lower levels of total protein, albumin, and globulin and higher levels of total bilirubin, bilirubin direct, bilirubin indirect, aspartate amino transferase, alanine amino transferase, alkaline phosphate, and gamma-glutamyl transferase in comparison to the control group [42]. Cannabis causes hepatotoxicity as described by Borini et al. (2004) and Ewinget et al. (2019) due to cholestatic damage, which is supported by an increase in ALP. Cannabis is less likely to cause liver fibrosis, which could account for the remission of liver abnormalities [40, 43].

Reviewer #3: It is good study. If not out of scope please add a couple of para on the benefit of THC in reducing inflammation. You can also state a bit about CBD if that is okay. The benefit of this study is far from seen.

Thank You.
