# Supplementary material for: Health status outcome among cannabis addicts after treatment of addiction
Source: PLoS One. 2023 Nov 27;18(11):e0290730. doi: 10.1371/journal.pone.0290730 (PMC10681311; doi:10.1371/journal.pone.0290730)
Supplement: Attachment — Submitted filename: reply.docx [file pone.0290730.s003.docx]

PONE-D-23-02435R1

Health status outcome among cannabis addicts after treatment of addiction

PLOS ONE

Dear Dr. Yasmina Abd‐Elhakim
Academic Editor
PLOS ONE

Journal Requirements:

Please review your reference list to ensure that it is complete and correct. If you have cited papers that have been retracted, please include the rationale for doing so in the manuscript text, or remove these references and replace them with relevant current references. Any changes to the reference list should be mentioned in the rebuttal letter that accompanies your revised manuscript. If you need to cite a retracted article, indicate the article’s retracted status in the References list and also include a citation and full reference for the retraction notice.

Thank you, I agree

All references are revised and corrected.

**Additional Editor Comments:**

It is highly recommended to attach a separate file of response to reviewer comments.

Thank you, I agree

Reviewers' comments:

Reviewer's Responses to Questions

**Comments to the Author**

1. If the authors have adequately addressed your comments raised in a previous round of review and you feel that this manuscript is now acceptable for publication, you may indicate that here to bypass the “Comments to the Author” section, enter your conflict of interest statement in the “Confidential to Editor” section, and submit your "Accept" recommendation.

Reviewer #2: (No Response)

Thank You,

My reply to your previous round comments:

Reviewer #2: This manuscript describes a study that evaluated changes in health status before and after treatment using body mass index, glycaemic status, liver function, renal function, and oxidative stress in cannabis addicts.
The study was carried out on 100 hospitalized patients receiving addiction treatment and 20 healthy volunteers. Pre- and post-treatment health status indicators were determined by using cannabis level in urine, Body Mass Index, vital signs, some biochemical and oxidative stress parameters. While no change was observed in the BMI values and oxidative stress parameters between the groups before and after the treatment, it was revealed that there was a significant decrease in biochemical parameters in the dependent group after the treatment. Current findings reveal that cannabis addiction damages various body systems and has significant effects on the health status of the addict. This is an interesting finding that could lead to further research, confirming the phenomenon and attempting to identify the cause.
It is seen that the differences in the data obtained especially in the discussion section of the article are not fully revealed. The data obtained while writing the discussion and articles about it were written. However, as the last sentence, the author's opinion on why this result may have occurred has not been added. For this reason, I think that eliminating the deficiencies given below in detail will be important for the quality of the article.
Overall the study appears to have been conducted appropriately and results interpreted correctly (with some exceptions mentioned below). The article reads well, but there are some deficiencies that need to be edited from beginning to end, as there are places that cause misunderstanding at some points. The fact that this study lasted for two years raises fundamental concerns about the rationale for this study. Specific concerns that need to be addressed are as follows:

1. While it was stated that the dependent group was selected from male members, gender was not specified in the control group.

Thank you, I agree

We did in the methodology section.

2. It should be scientifically stated why a two-year period was chosen to continue this study.

Thank you, I agree

Two-year period, to be sure of weaning from cannabis use

3. It was stated that the samples were collected both at the time of application and after the treatment. It is a matter of curiosity whether any measures have been taken to prevent this interval (approximately 2 years) from causing differences in the test results.

Thank you, I agree

The sample withdrawn immediately after finishing the treatment, after that the patient under the follow up period.

4. In particular, how to distinguish whether the differences in the results obtained from the evaluation methods used in the material method section over the years are due to the treatment or to normal physiological processes.

Thank you, I agree

The sample withdrawn immediately after finishing the treatment.
5. "Males were more likely than females to abuse substances, and most began using substances in high school." How did you come to this conclusion?

Thank you, I agree

This conclusion was obtained from previous studies and we delete it.

6. "Compared to the control group, current results do not show a significant reduction in BMI at admission and after treatment." Can you explain why?

Thank you, I agree

Energy uptake, storage, and conservation are primarily mediated by CB1 receptors. Through a number of techniques, it works to maximize energy uptake and conservation. Food is made more palatable by modulating taste and smell pathways by stimulating CB1 receptors [33]. There is a lot of proof that THC or cannabis use causes the CB1R to be downregulated. Regular cannabis usage is linked to CB1R downregulation and desensitization, and CB1R levels stay low for 3–4 weeks after use has stopped [34]. During this time, recently abstinent users would have decreased hunger and elevated metabolic rates. They won't, however, continue to have short-term stimulation of appetite, increased energy consumption, and decreased metabolic rates. Therefore, until CB1R levels reach those from before cannabis usage, weight won't rise because energy intake and storage are still suppressed and metabolism is increased [35]. Ngueta et al., 2015 reported that a higher metabolic rate in marijuana users explain the lack of association between cannabis use and BMI, despite a high energy diet [36]. These data explained the findings of the current study. As compared to the control group, the current results do not indicate a significant reduction in BMI at admission and after treatment.

7. Could you indicate the reason why the vital signs in Group II were significantly lower in the discussion section?

Thank you, I agree

Immediately following marijuana use, the heart rate and blood pressure may increase [35]. Increased sympathetic nervous system activity is responsible for the increased heart rate, supine systolic and diastolic blood pressures, and forearm blood flow caused by marijuana cigarettes [36]. After a few repeated dosages, tolerance to several of these effects becomes noticeable. After just a day or two of repeated exposure, there may be significant tolerance to changes in blood pressure and elevated heart rate. Tolerance is lost just as quickly when marijuana or THC use is discontinued. Blood pressure falls and the resting heart rate drops after a few days of frequent oral THC exposure or a marijuana plant extract [37].

8. It is predicted that the fluctuation in blood glucose level occurs.

Thank you, I agree

The present study demonstrated that, compared to the control group, there was a significant increase in the mean fasting blood glucose level values of cannabis user group at admission but no significant change in cannabis user group after treatment. After treatment, the mean fasting blood glucose levels in cannabis user group decreased significantly compared to their respective at admission values. Marijuana use was linked to lower levels of fasting insulin and homeostasis model assessment of insulin resistance [37]. Cannabis use for 12 months is linked to nearly double the risk of diabetic ketoacidosis in one study of adults with T1D when compared to no cannabis use [38]. The variations in cannabis strain potencies may help to explain the variations in blood glucose levels. The potent preparations ('ganja' and 'charas') suppress appetite whereas the weak preparation ('bhang') induces anorexia. THC levels in peripheral blood in people who have smoked one or more marijuana cigarettes have exceeded the range of 5 to 250 ng/ml, whereas intravenous delivery of 10 mg/kg THC produced blood levels of 720 ng/ml after 20 minutes [9]. Other research has demonstrated that cannabis influence the stress response and increase the release of adrenocorticotropin (ACTH) [39, 40].

9. “Our findings found that in comparison to the control group, there was a significant increase in the mean levels of albumin, total bilirubin, direct bilirubin, AST, ALT, and ALP in group II at admission, but no significant change in group II after treatment”. Please explain why this situation has arisen.

Thank you, I agree

Hepatotoxicity is a possible side effect of using illegal drugs, such as marijuana. A study by Borini et al. evaluating clinical and laboratory changes in chronic marijuana users showed about fifty percent of patients have enlarged spleen or liver or both. Also, marijuana use has been linked to an increase in serum levels of AST, ALT, and AP, regardless of the amount or duration of use [40]. Our findings found that in comparison to the control group, there was a significant increase in the mean levels of albumin, total bilirubin, direct bilirubin, AST, ALT, and ALP in cannabis user group at admission, but no significant change in cannabis user group after treatment. These findings are consistent with a study by Devinsky et al. from 2018 which discovered that liver enzymes increased in chronic cannabis users and returned to normal following the drug's administration [41]. Additionally, Wani et al., 2017 found that cannabis users had lower levels of total protein, albumin, and globulin and higher levels of total bilirubin, bilirubin direct, bilirubin indirect, aspartate amino transferase, alanine amino transferase, alkaline phosphate, and gamma-glutamyl transferase in comparison to the control group [42]. Cannabis causes hepatotoxicity as described by Borini et al. (2004) and Ewinget et al. (2019) due to cholestatic damage, which is supported by an increase in ALP. Cannabis is less likely to cause liver fibrosis, which could account for the remission of liver abnormalities [40, 43].

Reviewer #3: All comments have been addressed.

Thank you

2. Is the manuscript technically sound, and do the data support the conclusions?

The manuscript must describe a technically sound piece of scientific research with data that supports the conclusions. Experiments must have been conducted rigorously, with appropriate controls, replication, and sample sizes. The conclusions must be drawn appropriately based on the data presented.

Reviewer #2: (No Response)

The current findings show that cannabis addiction harms various body organs except the kidneys and seriously affects addicts' health. This study revealed that after treatment, there was no improvement in oxidative stress biomarker values, but there was an improvement in the other biological parameters.

Reviewer #3: Yes

Thank you

3. Has the statistical analysis been performed appropriately and rigorously?

Reviewer #2: (No Response)

Data were collected, tabulated, and statistically analyzed using the paired samples t-test to determine whether there were any significant differences between the data of the cannabis user group before and after treatment. Results will be expressed as means ± SD, and statistical significance will be determined if the p-value is less than 0.05. In addition, Pearson correlation coefficients were used to determine the relationships between the CWAS, THC levels, age, and duration of addiction with BMI, vital signs, renal, and hepatic parameters. The SPSS Version 21.0 [SPSS, Inc., Chicago] package will be utilized for all statistical analyses.

Please see the result section for our findings. It is revised and reformulated.

Reviewer #3: Yes

4. Have the authors made all data underlying the findings in their manuscript fully available?

The [PLOS Data policy](http://www.plosone.org/static/policies.action#sharing) requires authors to make all data underlying the findings described in their manuscript fully available without restriction, with rare exception (please refer to the Data Availability Statement in the manuscript PDF file). The data should be provided as part of the manuscript or its supporting information, or deposited to a public repository. For example, in addition to summary statistics, the data points behind means, medians and variance measures should be available. If there are restrictions on publicly sharing data—e.g. participant privacy or use of data from a third party—those must be specified.

Reviewer #2: (No Response)

All data are present as a separate file named supporting information.

Reviewer #3: Yes

5. Is the manuscript presented in an intelligible fashion and written in standard English?

PLOS ONE does not copyedit accepted manuscripts, so the language in submitted articles must be clear, correct, and unambiguous. Any typographical or grammatical errors should be corrected at revision, so please note any specific errors here.

Reviewer #2: (No Response)

The article was paraphrased and revised (see certificate)

Reviewer #3: Yes

6. Review Comments to the Author

Please use the space provided to explain your answers to the questions above. You may also include additional comments for the author, including concerns about dual publication, research ethics, or publication ethics. (Please upload your review as an attachment if it exceeds 20,000 characters)

Reviewer #2: Since I could not reach the "author response" content, which includes the answers to the corrections I have written, I could not re-evaluate. for this reason, I need an "author response" that responds to each of my criticisms in a neat and sequential manner. Therefore, I requested a major revision again.

Dear Thanks for your comment and I am ready to answer any further comment.

Reviewer #3: The article is better now. The topic is quite relevant and from an area where such studies are not much common so an article like this is good study

Thank you

7. PLOS authors have the option to publish the peer review history of their article ([what does this mean?](https://journals.plos.org/plosone/s/editorial-and-peer-review-process#loc-peer-review-history)). If published, this will include your full peer review and any attached files.


If you choose “no”, your identity will remain anonymous but your review may still be made public.


**Do you want your identity to be public for this peer review?** For information about this choice, including consent withdrawal, please see our [Privacy Policy](https://www.plos.org/privacy-policy).

Reviewer #2: No

Reviewer #3: No
